# Clinical and morphological features of large-cell neuroendocrine carcinomas and small-cell lung carcinomas expressing the DLL3 and ASCL1 oncoproteins

**DOI:** 10.1590/1414-431X2023e12921

**Published:** 2023-12-22

**Authors:** T.G. Prieto, C.M. Baldavira, J. Machado-Rugolo, E.H.R. Olivieri, E.C.A. da Silva, V.G. Silva, A.M. Ab'Saber, T.Y. Takagaki, V.L. Capelozzi

**Affiliations:** 1 Laboratório de Genômica e Histomorfometria, Departamento de Patologia, Faculdade de Medicina, Universidade de São Paulo, São Paulo, SP, Brasil; 2 Centro de Avaliação de Tecnologia em Saúde, Hospital das Clínicas, Faculdade de Medicina, Universidade Estadual Paulista, Botucatu, SP, Brasil; 3 Centro Internacional de Pesquisa, AC Camargo Cancer Center, São Paulo, SP, Brasil; 4 Centro de Pesquisa em Oncologia Molecular, Hospital de Câncer de Barretos, Barretos, SP, Brasil; 5 Fundação Oncocentro do Estado de São Paulo, São Paulo, SP, Brasil; 6 Divisão de Pneumologia, Instituto do Coração, Faculdade de Medicina, Universidade de São Paulo, São Paulo, SP, Brasil

**Keywords:** Large-cell neuroendocrine carcinomas, Small-cell lung carcinomas, Immunohistochemistry, Digital analysis, Transmission electron microscopy, DLL3/ASCL1

## Abstract

Intratumoral similarities and differences between large-cell neuroendocrine carcinomas (LCNECs) and small-cell lung carcinomas (SCLCs) are determined partially by the Notch signaling pathway, which controls the switch from neuroendocrine to slight/non-neuroendocrine cell fate. LCNECs are divided into two subgroups according to genomic alterations: type I LCNECs exhibit a neuroendocrine profile characterized by achaete‐scute homolog 1 (ASCL1)^high^/delta-like protein 3 (DLL3)^high^/NOTCH^low^ and type II LCNECs show the pattern ASCL1^low^/DLL3^low^/NOTCH^high^. Here, we used immunohistochemistry, transmission electron microscopy, and digital analysis to examine the role of the Notch ligand DLL3 as an immunomarker of the neuroendocrine state and ASCL1 as a regulator of cell-cell interactions in SCLCs and LCNECs. High DLL3 and ASCL1 expression was associated with atypical submicroscopic characteristics involving nuclear size, chromatin arrangement, Golgi apparatus, and endoplasmic reticulum, and was characteristic of type I LCNECs with similarity to SCLCs, whereas low DLL3 and ASCL1 expression was found in both SCLCs and type II LCNECs. In patients diagnosed at an early stage who did not have metastasis and who underwent chemotherapy, DLL3^high^ and ASCL1^high^ SCLCs and type I LCNECs were associated with a better prognosis and a lower risk of death. The present findings suggested that DLL3/ASCL1 are potential therapeutic targets and prognostic indicators in patients with SCLCs or LCNECs.

## Introduction

Small-cell lung carcinomas (SCLCs) and large-cell neuroendocrine carcinomas (LCNECs) are aggressive types of lung cancer that account for 15 and 2-3% of all lung cancer cases, respectively. They mainly affect older people who are heavy smokers, and their 5-year survival rates are 15-25 and 5%, respectively (1). LCNECs and SCLCs are treated primarily by surgery and chemotherapy (in SCLCs); however, chemotherapy has limited efficacy in LCNEC, and effective treatment for this tumor type is lacking (2). High-grade neuroendocrine carcinomas include borderline cases between LCNEC and SCLC (3); therefore, it is important to examine the histologic (microscopic) and hierarchical (submicroscopic) features of these tumor types to elucidate the underlying mechanisms and facilitate their classification, as well as to identify novel therapeutic targets.

Work in murine models suggests that neuroendocrine stem cells of the terminal bronchioli have been postulated as the cellular origin of primary SCLC. However, primary SCLC probably originates from two distinct oncogenic pathways: from the NOTCH pathway or from neuroendocrine stem cells with mutual bi‐allelic TP53 and RB1 alteration (4,5). Meder et al. (6) identified a pathway driving the pathogenesis of the SCLC involving activation of the NOTCH target achaete‐scute homolog 1 (ASCL1) and its ligand delta-like protein 3 (DLL3) and canonical WNT‐signaling in the context of mutual bi‐allelic RB1 and TP53 lesions. ASCL1 is a transcription factor that is crucial for neuroendocrine differentiation and is expressed by pulmonary neuroendocrine cells in SCLC (7). DLL3 is an inhibitor of Notch signaling during embryogenesis and functions in localizing NOTCH receptors in the Golgi apparatus (8). Stimulation of NOTCH receptors by DLL3 in the NOTCH signaling pathway promotes NOTCH cleavage. However, the activation of NOTCH signaling induces the transcriptional repressor Hes1, and Hes1 suppresses ASCL1 expression. These observations raised the possibility that the expression patterns of the NOTCH effector Hes1 might be involved in neuroendocrine cell differentiation (9). The negative feedback loop results in NOTCH^low^/DLL3^high^ cells and NOTCH^high^/DLL3^low^ cells (10).

Recently, George et al. (11) identified two molecular subgroups of LCNECs: type I LCNECs, which have a neuroendocrine profile characterized by ASCL1^high^/DLL3^high^/NOTCH^low^, and type II LCNECs, which show the pattern ASCL1^low^/DLL3^low^/NOTCH^high^. Although they share genomic alterations with non-small cell lung carcinoma, type I LCNECs and SCLCs exhibit a similar neuroendocrine profile, whereas type II LCNECs and SCLCs show genetic similarities. However, these tumors are distinguishable from SCLCs because of their reduced levels of neuroendocrine markers (11).

DLL3 mRNA and protein expression are detected in >80% of SCLC tumors, and both cytoplasmic and membranous staining is observed by immunohistochemistry (IHC) with a high level of homogeneity among malignant cells. However, in normal cells, DLL3 is expressed in a few cell types (e.g., neurons and pituitary cells) and located mainly in the cytoplasm (12). DLL3 expression is not limited to neuroendocrine lung tumors and is detected in other tumors of neuroendocrine origin including small-cell bladder cancer and melanoma (13).

DLL3 is considered a promising therapeutic target in SCLC, a malignancy with a low survival rate and for which effective treatments are lacking. Several DLL3-targeted agents are being evaluated in ongoing clinical studies of SCLC and other neuroendocrine tumors. DLL3-targeting modalities include antibody-drug conjugates, T-cell engagers, and chimeric antigen receptor (CAR) T cells (14).

Considering the limited therapeutic options for SCLC, it is important to identify markers of response and toxicity and to characterize DLL3 as a dynamic biomarker, in addition to the refinement of adverse event (AE) management approaches. These are important areas of development for DLL3-targeting therapies not only in SCLC but also in other neuroendocrine carcinomas (14).

The present study evaluated the expressions of DLL3 and ASCL1 in SCLC and LCNEC and their role in the diagnosis and prognosis of these tumors. In addition, we evaluated the correlation between the IHC staining pattern and the morphological and phenotypic characteristics of LCNECs and SCLCs and explored the similarities and differences in the expression of oncoproteins between these two tumor types.

## Material and Methods

### Study cohort

The surgical pathology files of the Clinicas Hospital and Heart Institute of the University of São Paulo, A. C. Camargo Cancer Center, and Hospital do Amor, in Barretos, São Paulo, were searched to identify patients with histologically confirmed LCNEC and SCLC diagnosed between January 1995 and December 31, 2017. Formalin-fixed paraffin-embedded (FFPE) samples from nine patients with surgically resected LCNEC (an orphan disease) and 42 patients with SCLC biopsies were found. Cases were reviewed by a thoracic pathologist (V.L.C.) to confirm the diagnosis and classified into SCLC and LCNEC according to the current World Health Organization criteria (1). Cases were staged according to the 8th edition of the AJCC/UICC staging (TNM classification) (15). Clinical information was obtained from medical records.

The internal ethics committees of all the participating institutions approved the study protocol (process number 1.077.100) with a waiver for informed consent by their review boards.

### Immunohistochemistry

FFPE tissue sections (3-μm-thick) were stained for IHC detection of CD56 (NCAM1, Santa Cruz, USA), synaptophysin (SYP, Abcam, USA), chromogranin A (CHGA, clone DAKA3, Agilent, USA), TTF-1 (NKX2-1, clone 8G7G3/1, Abcam), DLL3 (E3J5R, Cell Signaling, USA; 1:100 dilution), and ASCL1 (bs-1155R clone, Bioss, USA; 1:150 dilution). IHC for DLL3 and ASCL1 was performed using the Vectastain ABC detection system (Vector Laboratories, Inc., USA) after antigen retrieval (4×5 min in a microwave at 700 W). Immunoglobulin G (IgG) was used as the negative control to ensure the absence of nonspecific binding. For both biomarkers, positive cells were characterized by an inhomogeneous pattern that either obscured the nucleus, was located around the nucleus, or showed a punctate or diffuse expression in the cytoplasm.

### Semiautomated quantification

Slides containing SCLC and LCNEC samples were scanned using a Panoramic 250 full-slide scanner (3DHistech, Hungary) at 40× magnification. DLL3 and ASCL1 expression was quantified using QuPath software (version 0.2.1; Center for Cancer Research and Cell Biology, University of Edinburgh, Scotland), an open-source image analysis platform (16). Briefly, the quantification of samples in QuPath uses a simple automated and semi-assisted method. For each digitized slide, 5-10 non-coincident fields of analysis were selected, and the program automatically performed positive cell detection in each field. Positive cell detection was determined according to the results of immunostaining. The following patterns were observed: not homogeneous, obscuring the nucleus, surrounding the nucleus, and punctate or diffuse cytoplasmic expression. For cytoplasmic staining, Cytoplasm: DAB OD mean was used, and for nuclear staining, Nuclear: DAB OD mean was used. After quantification, QuPath-generated data included number of positive cells per mm^2^ of tissue and percentage of positive cells per mm^2^ of tissue. Although a biologically distinct cutoff point for DLL3 and ASCL1 expression has not been established, we used a DLL3 and ASCL1 H-score of 50% after modifications for patient classification according to previous clinical studies (17). The percentage of DLL3- and ASCL1-positive cells was determined, and DLL3 and ASCL1 expression was defined as high (≥50%) or low (<50%) to assess the correlation with clinicopathologic characteristics and patient outcome.

### Transmission electron microscopy (TEM)

Tissues were fixed in a 2% glutaraldehyde buffer and post-fixed in 1% OsO_4_. The samples were then washed with a 0.9% saline solution containing uranyl and sucrose overnight and soaked in Epon. Finally, the samples were stained with uranyl acetate and lead citrate and examined using a JEOL JEM-1010 electron microscope (USA).

### Statistical analysis

The chi-squared test or Fisher's exact test was used to evaluate the differences in categorical variables, and the Spearman correlation test was used to assess the correlations between variables. Overall survival (OS) was defined as the interval from the date of biopsy or surgical resection to the date of death. The Kaplan-Meier method was used to generate OS curves. The association between OS rate and other covariates was analyzed using the Cox proportional hazards model. The Statistical Package of Social Science (SPSS, IBM, USA) version 18 was used for all statistical analyses. Results with P<0.05 were considered statistically significant, and Bonferroni correction was used when necessary.

## Results

Table 1 lists the clinicopathological characteristics of the patients. The cohort included 51 patients diagnosed with high-grade neuroendocrine carcinomas (42 SCLCs and 9 LCNECs). The median age of the patients at diagnosis was 61 years, and there were 28 (54.9%) men and 23 (45.1%) women. Most patients were smokers (96.1%), and the majority were diagnosed at an advanced stage of the disease (86.3%). SCLC was located centrally, whereas LCNEC was located peripherally in the lung. There were 40 (78.4%) patients who received platinum-based chemotherapy and 14 (27.5%) who underwent brain radiotherapy. During the follow-up period, 20 (39.2%) patients developed systemic metastasis. The median follow-up period was 18 (0-60) months.

**Table 1:** Frequency of demographic and clinical-pathologic characteristics of patients with high grade neuroendocrine carcinomas.

Characteristics	Frequency (n, %)
Age (years), median and range	61 (24-83)
≥61	27 (52.9)
<61	24 (47.1)
Gender	
Male	28 (54.9)
Female	23 (45.1)
Tobacco history	
Yes	49 (96.1)
No	2 (3.9)
Histologic type	
Large cell neuroendocrine carcinoma	9 (17.6)
Small cell lung carcinoma	42 (82.4)
TNM stage^a†^	
Local	6 (11.8)
Advanced	44 (86.3)
Chemotherapy^a^	
Yes	40 (78.4)
No	6 (11.8)
Radiotherapy^a^	
Yes	14 (27.5)
No	31 (60.8)
Systemic metastasis^a^	
Yes	20 (39.2)
No	17 (33.6)
Follow-up (months)	18 (0-60)

^a^No clinical information: TNM stage (1), chemotherapy (5), radiotherapy (6), and systemic metastasis (14). ^†^8th International Association for the Study of Lung Cancer.

**Figure 1:**
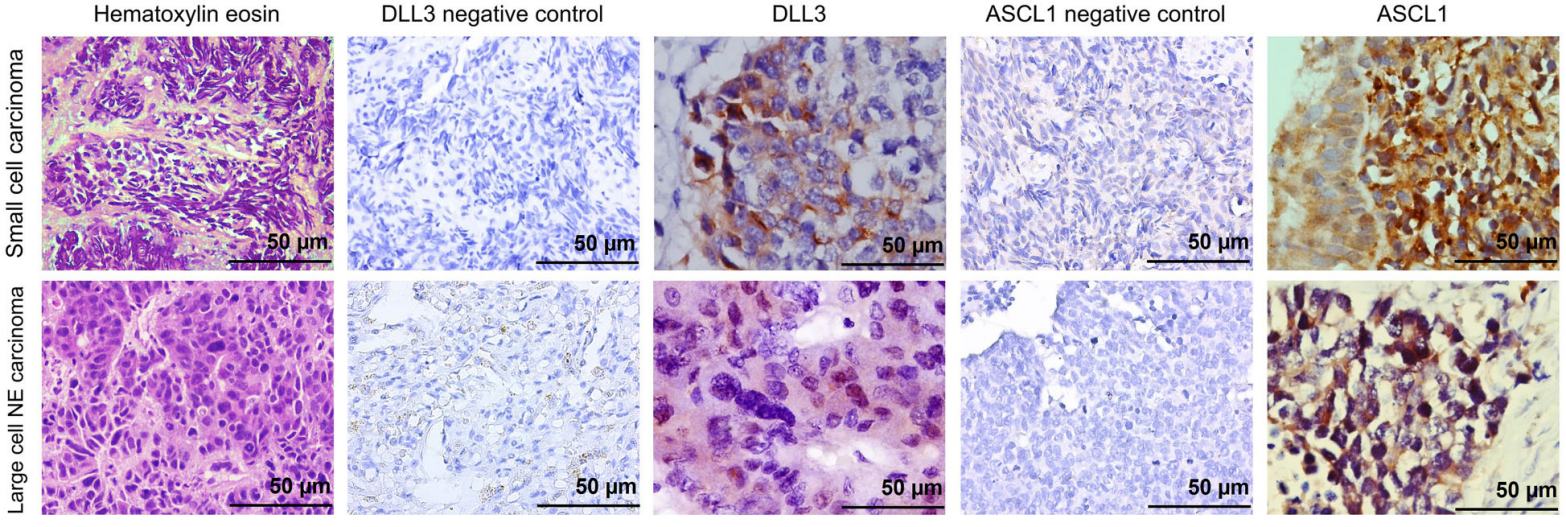
Hematoxylin-eosin, negative control, and immunohistochemistry (IHC) staining for DLL3 and ASCL1 in SCLCs and LCNECs (n=51). In viable areas without nuclear apoptosis, SCLCs were characterized by small cells with scant cytoplasm, ill-defined cell borders, finely granular nuclear chromatin, and small nucleoli. Neoplastic cells were round, oval, or spindle-shaped and showed cytoplasmic/membranous and perinuclear dot-like staining of DLL3 and nuclear expression of ASCL1. LCNECs showed characteristic organoid morphology, with nuclei larger than those of SCLCs, a prominent cytoplasm, nucleoli, and coarse chromatin. These cytologic features were highlighted by the perinuclear expression of DLL3 and the nuclear expression of ASCL1. Original magnification, 400× (hematoxylin-eosin and negative control) and 1000× (IHC); scale bar, 50 μm. DLL3: delta-like protein 3; ASCL1: achaete‐scute homolog 1; SCLC: small-cell lung cancer; LCNEC: large-cell neuroendocrine carcinoma.

**Figure 2:**
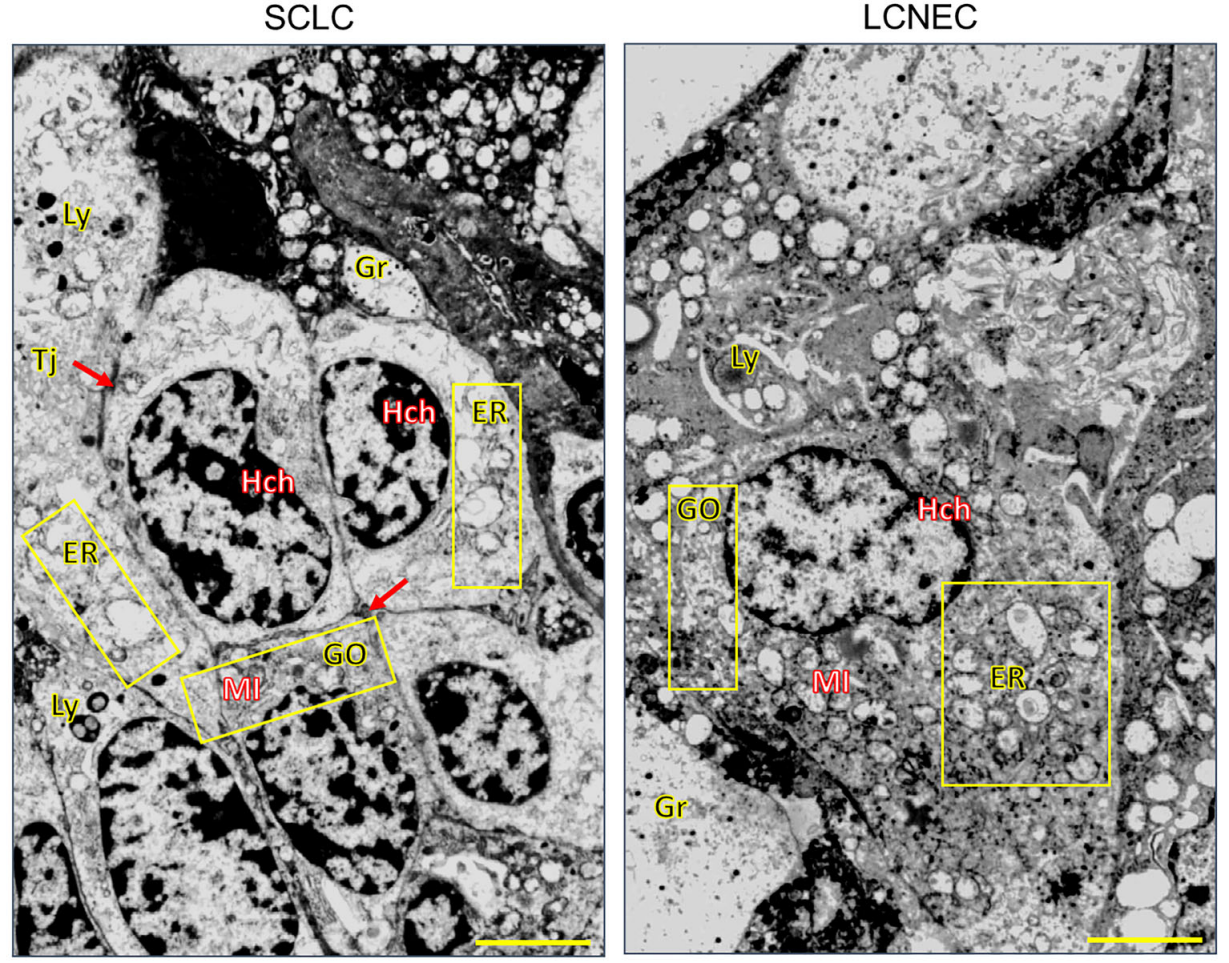
Transmission electron microscopy images of SCLC and LCNEC cells. SCLCs were composed of medium-sized round cells and the presence of cytoplasm with granules (Gr), ill-defined cell borders with tight junctions (Tj), finely granular nuclear heterochromatin (Hch) forming dots next to the nuclear membrane, and conspicuous nucleoli. LCNECs were also characterized by medium-sized round cells, larger nuclei than those of SCLCs with thin Hch, a prominent cytoplasm with Gr, small nucleoli, and nuclear heterochromatin forming peripheral aggregates at the nuclear membrane. In SCLCs, in addition to dilated cisternae of the Golgi (GO) apparatus and endoplasmic reticulum (ER), the cytoplasm shows characteristic inclusions that likely represent lysosomes (Ly). In LCNECs, the cytoplasm of cancer cells showed prominent dilated cisternae of the GO apparatus and ER and enlarged mitochondria (MI). These abnormal submicroscopic alterations may be associated with high levels of cytoplasmic/membranous DLL3 expression, perinuclear dot-like staining of DLL3, and nuclear expression of ASCL1. SCLC: small-cell lung carcinoma; LCNEC: large-cell neuroendocrine carcinoma; DLL3: delta-like protein 3; ASCL1: achaete‐scute homolog 1. Original magnification, 20,000×; scale bar, 2 µm.

Figure 1 shows the histological characterization of LCNECs and SCLCs stained by hematoxylin-eosin and the results of DLL3 and ASCL1 immunostaining, and Figure 2 shows the TEM results.

**Figure 3:**
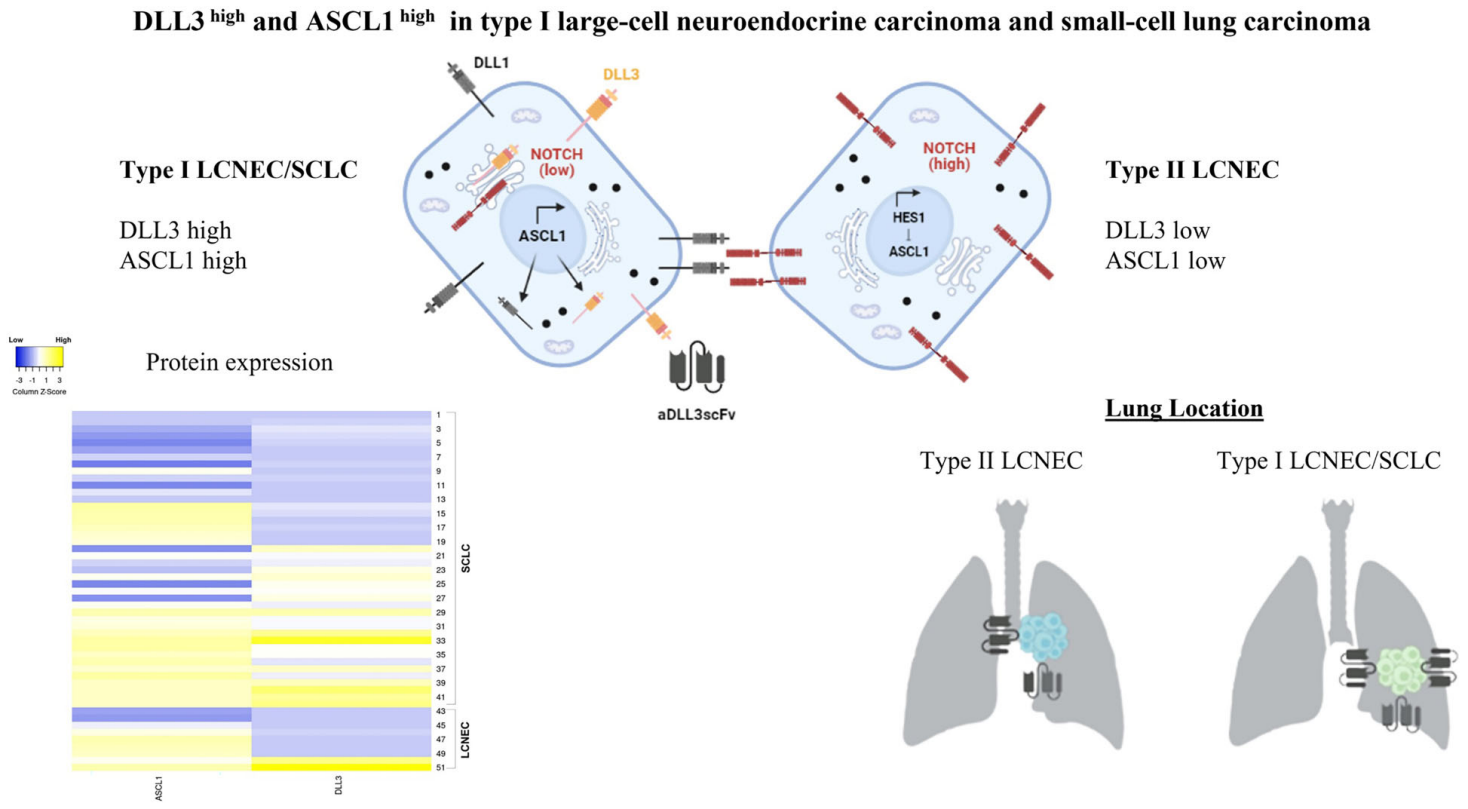
Representative heat map for high and low DLL3 and ASCL1 gradients in small-cell lung carcinomas (SCLCs) and large-cell neuroendocrine carcinomas (LCNECs). DLL3: delta-like protein 3; ASCL1: achaete‐scute homolog 1; HES1: transcriptional repressor.

Figure 3 shows a representative heat map of high and low DLL3 and ASCL1 gradients in LCNEC and SCLC. High (≥50%) DLL3 expression was detected in 39 patients; in almost all patients, DLL3 expression was found in 50-95%. Of the 39 DLL3-high patients, 34 had SCLC and 5 had LCNEC. DLL3 staining indicated a strong cytoplasmic expression pattern with perinuclear accentuation in 83.3% of SCLCs and 55.6% of LCNECs. ASCL1 expression was detected in the nucleus of tumor cells in all cases, with high expression (≥50%) in 76.2% of SCLCs and 77.8% of type I LCNECs. Low ASCL1 expression (<50%) was detected in 23.8% of SCLCs and 22.2% of LCNECs, whereas low DLL3 (<50%) was detected in 16.7% of SCLCs and 44.4% of LCNECs. Six patients were negative for DLL3 expression.

LCNEC and SCLC patients were divided into two groups (low and high expression) according to DLL3 and ASCL1 expression using the 50% cutoff point. LCNECs with the neuroendocrine profile ASCL1^high^/DLL3^high^/NOTCH^low^ were categorized as type I LCNECs, whereas ASCL1^low^/DLL3^low^/NOTCH^high^ cases were categorized as type II LCNECs.

High expression of DLL3 in SCLCs and LCNECs was associated with lymph node and distant metastasis (P=0.038 and P=0.028, respectively), and low DLL3 expression was more prevalent in patients without distant metastasis. DLL3 and ASCL1 expression was not significantly correlated with other clinicopathological features, whereas a statistically significant positive correlation between ASCL1 and DLL3 expression was observed (ρ=0.314 and P=0.03).

The morphological features of LCNEC and SCLC tumor cells were analyzed by TEM and optical microscopy (Figure 2). SCLCs were characterized by medium-sized round cells with cytoplasmic granules, ill-defined cell borders, tight junctions, finely granular nuclear chromatin and heterochromatin forming dots next to the nuclear membrane, and conspicuous nucleoli. LCNECs were characterized by medium-sized round cells, larger nuclei than those in SCLCs, a prominent cytoplasm with granules, small nucleoli, and nuclear heterochromatin forming clumps at the periphery enhancing the nuclear membrane. In SCLCs, in addition to dilated Golgi (GO) apparatus and endoplasmic reticulum (ER) cisternae, the cytoplasm showed inclusions resembling lysosomes. In LCNECs, the cytoplasm showed prominent dilated GO apparatus and ER cisternae and enlarged mitochondria. These submicroscopic alterations were associated with high cytoplasmic/membranous DLL3 expression, perinuclear dot-like DLL3 staining, and nuclear ASCL1 expression.

In the survival analysis, variables with a known impact on prognosis and/or with a P-value <0.2 in the univariate analysis were included in the multivariate Cox model as independent variables, whereas DLL3 and ASCL1 were co-dependent variables (Table 2). Although DLL3^high^ and ASCL1^high^ tumor expression was not an independent predictor in the multivariate analysis, after adjusting for early stage, absence of metastasis, and response to chemotherapy, patients with these characteristics had a better prognosis and a lower risk of death.

**Table 2:** Variables associated with risk of death for patients with high grade neuroendocrine carcinomas using univariate and multivariate Cox regression. Chi squared 12.57, P=0.028.

Clinical-pathologic characteristics	Univariate analysis^a^			Multivariate analysis^b^	
	HR (95%CI)	HR	P-value	HR (95%CI)	P-value
Age (years) median					
<61	1.03 (0.55-1.93)	0.038	0.904		
≥61 (reference)					
Gender					
Male	1.39 (0.75-2.60)	0.335	0.290		
Female (reference)					
Tobacco history					
Yes	3.37 (0.45-24.96)	1.215	0.234		
No (reference)					
Clinical stage^†^					
Local	0.55 (0.21-1.43)	-0.593	0.223	0.58 (0.06-5.36)	0.635
Advanced (reference)					
Lymph node status^†^					
N0	1.22 (0.36-4.06)	0.203	0.740		
N1 (reference)					
Systemic metastases status^†^					
M0	0.54 (0.26-1.15)	-0.602	0.112	0.36 (0.15-0.86)	**0.022**
M1 (reference)					
Chemotherapy					
Yes	0.21 (0.08-0.54)	-1.538	0.001	0.13 (0.03-0.50)	**0.003**
No (reference)					
Radiotherapy					
Yes	0.72 (0.36-1.43)	-0.324	0.356		
No (reference)					
Histo-protein category					
Type I LCNEC DDL3^low^ ASCL-1^low^	0.94 (0.48-1.82)	-0.063	0.85	2.19 (0.86-5.54)	0.105
Type II LCNEC DDL3^high^ ASCL-1^high^	1.74 (0.84-3.60)	0.553	0.14	0.48 (0.11-2.21)	0.079
SCLC DDL3^high^ ASCL-1^high^	1.73 (0.83-3.60)	0.553	0.137	0.56 (0.14-2.15)	0.061
SCLC DDL3^low^ ASCL-1^low^	0.68 (0.16-2.87)	-0.385	0.601	1.01 (0.98-1.03)	0.352

^a^Univariate analysis was performed without any adjustment to generate risk ratios with confidence intervals for individual risk for each of the survival parameters; ^b^Multivariate analysis was performed to analyze the effects of various risk parameters on survival. HR: hazard risk (β coefficient); CI: confidence interval. ^†^8th International Association for the Study of Lung Cancer.

## Discussion

In the current study, we performed a histologic and hierarchical analysis of LCNECs and SCLCs and found that type I LCNECs were markedly similar to SCLCs, both exhibiting the ASCL1^high^/DLL3^high^ neuroendocrine oncoprotein profile. As reported previously, LCNECs were stratified into two subgroups: type I LCNECs (containing STK11/KEAP1 alterations) and type II LCNECs (containing RB1 alterations) (11). Although type II LCNECs are genetically similar to SCLCs, certain distinct features such as decreased expression of neuroendocrine markers and high activity of the NOTCH pathway distinguish them from SCLCs (11).

More than 80% of SCLC tumors express DLL3 mRNA and protein. IHC shows cytoplasmic and membranous staining with high homogeneity among malignant cells (12). Hermans et al. (18) detected DLL3 expression in 74% of 94 advanced-stage LCNEC patients. The present results were consistent with those of Rudin et al. (17), who found high DLL3 expression in 74% of 39 biopsy samples from SCLCs and LCNECs. However, there is limited information about the IHC procedure used in these studies, the DLL3 and ASCL1 antibodies, the percentage of positive cells, and survival data.

SCLC has a low survival rate and effective treatments are lacking. DLL3, which shows surface expression specific to tumor cells, is thus an attractive therapeutic target, and DLL3-targeted therapies are currently under clinical investigation, with promising antitumor activity demonstrated to date (19).

Several agents are being explored in preclinical and clinical studies focusing on the development of novel targeted therapies. These newly developed agents include the bispecific T-cell engager (TCE) tarlatamab and other TCEs such as HPN328, BI 764532, QLS31904, RO7616789, and PT217, as well as the chimeric antigen receptor (CAR) T-cell therapy AMG 119 (19).

The data showing that DLL3 is upregulated in high-grade neuroendocrine neoplasms (NENs) and associated with worse clinical outcomes support the potential of DLL3-targeting agents for the treatment of these challenging tumors, and some of these agents have already demonstrated clinical antitumor activity.

In the present cohort, we found differences in the expression of DLL3 between SCLCs and LCNECs. However, regardless of their distinct histomorphological features, certain similarities between them were observed in the TEM examination.

Similarities in cell size, nuclei, and heterochromatin characteristics between SCLCs and type I LCNECs were detected by TEM. Organelle changes such as dilated cisternae of the GO apparatus and ER, enlarged mitochondria, and cytoplasmic inclusions typical of lysosomes were observed in both neuroendocrine carcinomas. These abnormal submicroscopic features are suggestive of the activation of NOTCH receptors in the GO apparatus by the DLL3 ligand in the NOTCH signaling pathway (20), which is an inhibitor of ASCL1 (9). The negative feedback loop results in the generation of NOTCH^low^/DLL3^high^ cells and NOTCH^high^/DLL3^low^ cells (10). This altered phenotype and oncoprotein profile may be associated with high cytoplasmic/membranous expression of DLL3, perinuclear dot-like staining of DLL3, and nuclear expression of ASCL1.

Although the Cox multivariate regression analysis indicated that ASCL1 and DLL3 expression was not an independent prognostic factor, patients diagnosed at an early stage, without metastasis, who were treated with chemotherapy, and who had DLL3^high^ and ASCL1^high^ tumors had a better prognosis with a lower risk of death.

Overall, we provided a characterization of neuroendocrine lung tumors that integrated morphology, phenotype, hierarchical profile, and neuroendocrine oncoprotein expression. The results suggested that defining the differences between type I or type II LCNECs and SCLCs is important to predict the response to treatment and to further understand the morphological ontogeny in lung cancer patients.
